# Seroprevalence of hepatitis C virus markers in multi-transfused children with beta-thalassemia

**DOI:** 10.1186/1471-2334-12-S1-P42

**Published:** 2012-05-04

**Authors:** Kiran Madhusudhan, SP Thyagarajan

**Affiliations:** 1 Shree Balaji Medical College & Hospital, Chennai, India; 2 Sri Ramachandra Medical College & Research Institute, Chennai, India

## Background

To study the seroprevalence of hepatitis C virus in multi-transfused children with β-thalassemia and compared with non transfused children and healthy controls. β-thalassemic children fail to thrive, with growth and developmental retardation and suffer microcytic hypochromic anemia. Since regular blood transfusions are given to maintain haemoglobin at a safe level, these children are at a high risk of acquiring hepatitis C virus through transfusions.

## Methods

Study group, consisted of children 2-13 years with β-thalassemia and received more than 5 transfusions. Matched control group consisted of 30 children with β-thalassemia and no transfusion. Control group, consisted of 30 normal healthy children serum samples from all three groups were tested for antibodies to hepatitis C virus using commercial ELISA kits.

## Results

Study showed 32% anti H hepatitis C virus positivity in multi-transfused and 0% in matched and healthy control groups. Hepatitis C virus infection showed a significant increase in relation to the number of transfusions received.

## Conclusion

This observation is of great concern, as these children are at a risk of developing chronic hepatitis, cirrhosis and hepatocellular carcinoma. Since vaccination against hepatitis C virus is not available, highly sensitive and specific screening methods of donor blood in blood banks must be made mandatory.

